# Frailty and associated healthcare expenditures among patients undergoing total hip and knee arthroplasty^☆^

**DOI:** 10.1016/j.tjfa.2025.100030

**Published:** 2025-03-05

**Authors:** Donna Ron, Alton B. Daley, Marcus P. Coe, Michael D. Herrick, Robert H. Roth, Alexander T. Abess, Pablo Martinez-Camblor, Stacie G. Deiner, Myles D. Boone

**Affiliations:** a Department of Community and Family Medicine, Dartmouth Hitchcock Medical Center, Lebanon, NH, Geisel School of Medicine at Dartmouth, Hanover, NH; b Department of Anesthesiology, Critical Care, and Pain Medicine, Meir Medical Center and Tel Aviv University, 59 Tchernichovsky St, Kefar Sava 4428164, Israel; c Department of Orthopaedic Surgery, Dartmouth Hitchcock Medical Center, Lebanon, NH, Geisel School of Medicine at Dartmouth, Hanover, NH; d Department of Anesthesiology and Perioperative Medicine, Dartmouth Hitchcock Medical Center, Lebanon, NH, Geisel School of Medicine at Dartmouth, Hanover, NH; e Department of Biomedical Data Science, Geisel School of Medicine at Dartmouth, Hanover, NH; f Department of Neurology, Dartmouth Hitchcock Medical Center, Lebanon, NH, Geisel School of Medicine at Dartmouth, Hanover, NH

**Keywords:** Frailty, Health expenditures, Arthroplasty, Medicare

## Abstract

**Background:**

Major joint surgery is one of the largest components of Medicare spending in the US and the most frequent major procedure performed in older adults. Increasing age is associated with increasing prevalence of frailty, but the influence of frailty on healthcare expenditures following arthroplasty has yet to be adequately explored.

**Objective:**

To explore the association between frailty and healthcare expenditures in the year following total hip and knee arthroplasties.

**Design:**

Retrospective cohort study

**Setting:**

United States population

**Participants:**

Medicare beneficiaries 65 and older undergoing total knee or hip arthroplasty (*n* = 1,152,872) from 2017 through 2018.

**Measurements:**

Claims-based frailty index (exposure), total 1-year Medicare expenditures broken down by category (primary outcome), in-hospital complications, length of stay, discharge destination, readmission and mortality (secondary outcomes).

**Results:**

Among 435,496 patients who underwent hip (37.8 %) and 717,376 patients who underwent knee arthroplasty (62.2 %), the mean age was 73.7 years and 19.2 % were classified as frail. Median total expenditures in US dollars at one year were higher in those with frailty ($247,503; IQR [$169,400-$391,176]) relative to the prefrail ($179,379 [$127,396-$265,039]) and robust ($130,314 [$85,438-$199,605]) groups. Total expenditures included the index surgical admission, rehospitalization, skilled nursing care, and outpatient care, all of which were higher with increasing frailty. However, the surgical procedure accounted for less than a third of the total 1-year healthcare costs and was the category with the lowest degree of variation between patients. Frailty was also associated with longer lengths of stay, higher risks of complications, readmission, and mortality and lower likelihood of being discharged home after the procedure.

**Conclusions:**

Among older adults undergoing total hip and knee arthroplasty, frailty is associated with higher healthcare expenditures, predominantly driven by longitudinal care during the year following the procedure. More research is needed to test interventions to improve outcomes and reduce cost in this high-risk population.

## 1 Introduction

Major joint arthroplasty is the most common elective major surgical procedure among older adults [1]. Joint surgery is also responsible for the greatest proportion of Medicare spending in the United States [[2], [3], [4]]. Increasing age is associated with increasing prevalence of frailty, a syndrome of vulnerability and/or health deficit accumulation associated with increased susceptibility to adverse outcomes when confronted with a stressor [5]. Frailty is common among patients undergoing arthroplasty [6], and has been linked with longer hospital lengths of stay and increased risk of mortality, readmission, postoperative complications and institutionalization [[7], [8], [9], [10]]. However, frailty appears to be at least to some extent a reversible process [[11], [12], [13], [14], [15]]. This may be especially relevant for patients undergoing arthroplasty since these procedures often reduce debilitating pain, resulting in improved joint function and mobility, reducing fall risk and enabling increased physical activity [11,16].

Previous studies have looked at hospital costs associated with frailty among surgical patients [7,17], but few have focused on patients undergoing total hip and knee arthroplasty [18,19], and the total healthcare expenditures over the course of the year following the procedure have yet to be explored. We hypothesized that among patients undergoing hip and knee total joint arthroplasty (TJA), those with increasing frailty would incur higher healthcare expenditures over the course of the year following the procedure, and this association would remain significant when adjusting for demographic and social factors.

To examine the postoperative outcomes and expenditures among older adults undergoing TJA, we conducted a retrospective cohort study using Medicare claims data. Our primary objective was to determine whether there is an independent association between frailty and cost, as well as to identify the major drivers of increased cost in this population. By addressing this gap in the literature, we hope to aid in the development and refinement of value-based payment systems and targeted interventions to improve outcomes for older surgical patients.

## 2 Methods

This study was approved by the Dartmouth College Committee for the Protection of Human Subjects institutional review board. Informed consent was waived owing to secondary research on administrative data previously collected, per institutional policy. The reporting of this study was conducted in accordance with the Strengthening the Reporting of Observational Studies in Epidemiology (STROBE) reporting guideline for cohort studies.

### 2.1 Data sources and study population

This was a retrospective cohort study using Centers for Medicare & Medicaid Services (CMS) claims data for community dwelling US Medicare beneficiaries aged 65 years and older who underwent an elective total hip or knee arthroplasty between Jan 1, 2017, and Dec 31, 2018. CMS data files included the 100 % sample Medicare provider analysis and review file, outpatient, carrier, and master beneficiary summary files. To identify patients who underwent total hip and knee arthroplasty, we filtered surgical procedures based on International Statistical Classification of Diseases, 10th Revision, Clinical Modification (ICD-10-CM) codes. The procedural codes we used for total hip and knee surgery are listed in **Supplemental Table 1**. We defined an elective case based on admission source, and only included patients who presented from home; those who presented from the Emergency Room or Skilled Nursing Facilities were excluded from the analysis (**Supplemental Fig. 1**).

### 2.2 Exposure

We measured frailty using the Claims Based Frailty Index (CBFI) [20,21]. The CBFI is a validated measure of frailty that utilizes administrative data (ICD-10 codes, Current Procedural Terminology (CPT) codes, and Healthcare Common Procedure Coding System (HCPCS) codes) to predict the risk of adverse health outcomes and increased healthcare utilization. We calculated a frailty index (range of 0–1 with higher values indicating more frailty) for each beneficiary using publicly available programming codes (https://dataverse.harvard.edu/dataverse/cfi) and categorized them according to data-driven criteria to rationally distribute frailty as robust (0–0.12), prefrail (0.12–0.20) and frail (>0.20). We captured administrative data with a three-year “lookback” from the date of the index surgical procedure to calculate the score.

### 2.3 Outcomes

The primary outcome was total Medicare payments within 1 year following the procedure. These payments represented the sum of costs associated with the primary admission for the surgical procedure, readmissions, skilled nursing facilities (SNF), home health care, long-term care, physician office visits, and radiological or laboratory tests. Secondary outcomes included inpatient complications (e.g., stroke, myocardial infarction, pulmonary embolism, infection); discharge destination (home, rehabilitation, SNF); length of stay, 30-day and 90-day readmission and one-year mortality.

### 2.4 Analysis

Continuous and categorical variables were described as mean and standard deviations, and with absolute and relative frequencies. Demographic characteristics and comorbidities were compared across frailty categories using *Chi*-square and Welch tests as appropriate. A complete case approach was used to address missing data in the primary analysis. No imputation for missing data was performed.

Based on the asymmetric distribution of payment data, and in order to explore different behaviors at different payment levels, we selected a non-parametric quantile regression model to estimate the unadjusted and adjusted association of frailty with Medicare payments. We created adjusted models using *a priori* defined patient and hospital characteristics. These included age, gender, race, Social Deprivation Index (SDI) [22], and hospital location (rural or urban). We also adjusted for days alive in the first year to account for missed opportunities for Medicare payments. The SDI uses demographic information to estimate the effect of socio-economic disparities on health outcomes [23]. We included an additional model adjusting for Hierarchical Condition Categories (HCC) in addition to the aforementioned variables to examine the degree to which increased costs may be expected versus unexpected based on the higher comorbidity burden among patients who are more frail. The HCC is a risk-adjustment model developed by CMS to estimate future Medicare payments. To more easily demonstrate the cost associated with a unit change in frailty, we transformed frailty (scale 0–1) to “relative frailty” (RF) which was defined as RF= 100×(Frailty - m)/(M - m) where m and M represent the minimum and maximum values in the cohort, respectively. Results of the final models are presented as our primary analysis and are reported as β estimates and their associated 95 % CIs. We modeled payment data for 1) index surgical procedure 2) Readmission (days 1–30) 3) Inpatient hospitalization (days 31–365) 4) Outpatient costs 5) Skilled Nursing Care and 6) Total costs. To explore the uniformity of payments based on relative frailty, we calculated Gini indices for each class of payment. In addition, we conducted a prespecified sensitivity analysis to estimate the frailty and payment association as a function of type of arthroplasty (hip versus knee). All models contained the same variables as in the adjusted primary outcome.

## 3 Results

Among 1,152,872 Medicare beneficiaries who underwent a total hip (435,496 patients; 37.8 %) or total knee arthroplasty (717,376 patients, 62.2 %), the mean age [SD] was 73.7 [6.2] years; 62.2 % were women and 6.0 % were Black, 1.1 % were Hispanic and 88.6 % were White.

### 3.1 Baseline characteristics based on frailty status

To describe the characteristics of more versus less frail patients, based on the observed distribution of the frailty index, 385,700 patients (33.5 %) were classified as robust (mean frailty score [SD] 0.104 [0.01]), 546,322 patients (47.4 %) as prefrail (mean frailty score [SD] 0.157 [0.02]) and 220,850 patients (19.2 %) as frail (mean frailty score [SD] 0.245 [0.04]). Compared with prefrail and robust patients, patients with frailty were older (mean age [SD] 75.9 years [6.8], 73.1 [6.0], 73.4 [5.8]), a greater proportion were women (%: 71.0, 61.5, 58.4), they had more baseline comorbidities (mean [SD] Hierarchal Condition Category score, 1.91 [1.21], 0.47 [0.70], 0.35 [0.63]), and a higher social deprivation index (mean (SD) 40.3 [25.9], 37.4 [25.7], 39.1 [26.4]) (Table 1). Patients with frailty were more likely to have a diagnosis of dementia (n [%]: 25,497 [11.5]; 2,471 [0.5]; 13 [0]) and a larger proportion of them underwent total hip arthroplasty relative to those who were prefrail or robust (n [%]: 97,118 [44.0]; 199,937 [36.6]; 138,441 [35.9]).

**Table 1:** Baseline Patient Characteristics by Frailty Category^a^.

	Robust (*n* = 385,700)		Prefrail (*n* = 546,322)		Frail (*n* = 220,850)			
	N/mean	%/SD	N/mean	%/SD	N/mean	%/SD	p-value	SMD^b^
Frailty score	0.104	0.01	0.157	0.02	0.245	0.04	<0.001	−4.782
Hip Arthroplasty	138,441	35.9	199,937	36.6	97,118	44.0	<0.001	−0.166
Knee Arthroplasty	247,259	64.1	346,385	63.4	123,732	56.0	<0.001	0.166
Age in years	73.4	5.82	73.1	5.96	75.9	6.79	<0.001	−0.390
Female sex	225,107	58.4	335,739	61.5	156,765	71.0	<0.001	−0.266
Race								
Asian	3400	0.88	4687	0.86	1474	0.67	<0.001	0.024
Black	25,418	6.59	31,489	5.76	12,038	5.45	<0.001	0.048
Hispanic	5451	1.41	4995	0.91	2135	0.97	<0.001	0.041
North American Native	671	0.17	2130	0.39	1067	0.48	<0.001	−0.054
White	337,183	87.4	484,536	88.7	200,009	90.6	<0.001	−0.101
Other	5234	1.36	6435	1.18	1800	0.82	<0.001	0.052
Unknown	8343	2.16	12,050	2.21	2327	1.05	<0.001	0.088
Region								
Midwest	117,411	30.4	180,998	33.1	72,507	32.8	<0.001	−0.051
Northeast	71,975	18.7	97,106	17.8	36,453	16.5	<0.001	0.057
South	99,883	25.9	159,514	29.2	71,056	32.2	<0.001	−0.139
West	87,709	22.7	100,154	18.3	38,004	17.2	<0.001	0.139
Outside Continental US	8722	2.26	8550	1.57	2830	1.28	<0.001	0.074
Rural hospital	27,447	7.1	57,354	10.5	23,540	10.7	<0.001	−0.125
Social Deprivation Index	39.1	26.4	37.4	25.7	40.3	25.9	<0.001	−0.047
Dementia	13	0.00	2471	0.45	25,497	11.5	<0.001	−0.511
HCC Score	0.35	0.63	0.47	0.70	1.19	1.21	<0.001	−0.866

a Based on claims-based frailty index and categorized into robust (0–0.12), prefrail (0.12–0.20) and frail (>0.20).

b SMD for robust – frail Abbreviations: SD= Standard deviation, SMD= Standardized mean difference, HCC= Hierarchical Condition Category.

### 3.2 Unadjusted and adjusted payment outcomes

**Fig. 1:**
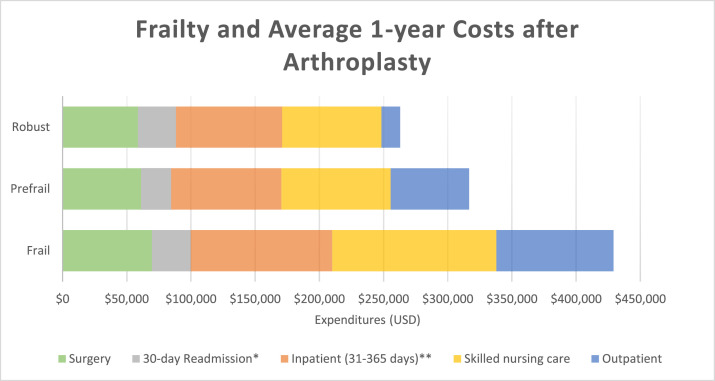
Frailty and Average 1-year Expenditures after Total Hip or Total Knee Arthroplasty *30-day readmission costs among patients who were readmitted **Inpatient costs among patients readmitted 31–365 days after the procedure.

Total Medicare payments at one year were higher in those with frailty relative to the prefrail and robust groups (mean cost in USD [SD]: 337,800 [312,989], 228,857 [193,974], 169,065 [161,483], median [Q1-Q3]: 247,503 [169,400–391,176], 179,379 [127,396–265,039], 130,314 [85,438–199,605]) (Table 2). Total payments included payments related to the index surgical procedure, payments related to 30-day readmission, inpatient care incurred between 31 and 365 days after surgery, skilled nursing care, and outpatient care, all of which were higher with increasing frailty (Fig. 1). The index surgical procedure accounted for 35 %, 27 % and 21 % of the total 1-year expenditures for the robust, prefrail, and frail groups, respectively.

**Table 2:** Frailty^a^, Outcomes and Expenditures (unadjusted).

	Robust (*n* = 385,700)		Prefrail (*n* = 546,322)		Frail (*n* = 220,850)			
	N/mean	%/SD	N/mean	%/SD	N/mean	%/SD	p-value	SMD^b^
**Outcomes**								
Length of Stay (days)	2.1	1.4	2.2	1.2	2.8	1.95	<0.001	−0.431
30-day Readmission	15,029	3.90	23,097	4.23	23,167	10.5	<0.001	−0.257
90-day Readmission	29,438	7.63	43,443	7.95	37,802	17.1	<0.001	−0.291
30-day Mortality	595	0.15	619	0.11	1101	0.50	<0.001	−0.060
Mortality (1 year)	4418	1.15	4949	0.91	8132	3.68	<0.001	−0.166
**ICU stay**	10,154	2.63	13,320	2.44	11,480	5.20	<0.001	−0.133
**Discharge destination**								
Home	314,221	81.5	438,395	80.2	126,515	57.3	<0.001	0.544
SNF	31,281	8.11	42,344	7.75	36,574	16.6	<0.001	−0.259
Rehabilitation	6060	1.57	16,011	2.93	14,962	6.77	<0.001	−0.262
Other	34,138	8.85	49,572	9.07	42,799	19.4	<0.001	−0.306
**Hospital Complications**								
Delirium	1142	0.30	615	0.11	3126	1.42	<0.001	−0.122
Pneumonia	668	0.17	830	0.15	1461	0.66	<0.001	−0.076
Pulmonary Embolism	629	0.16	923	0.17	561	0.25	<0.001	−0.020
DVT	909	0.24	1344	0.25	1048	0.47	<0.001	−0.040
Stroke	4137	1.07	5276	0.97	6604	2.99	<0.001	−0.136
Cardiac Arrest	62	0.02	84	0.02	94	0.04	<0.001	−0.015
Acute MI	409	0.11	438	0.08	758	0.34	<0.001	−0.050
Renal Insufficiency	48,361	12.5	64,168	11.8	54,780	24.8	<0.001	−0.319
Wound Infection	11	0.00	28	0.01	24	0.01	<0.001	−0.010
SSI	83	0.02	88	0.02	118	0.05	<0.001	−0.016
UTI	3746	0.97	5225	0.96	6091	2.76	<0.001	−0.132
Sepsis	240	0.06	214	0.04	509	0.23	<0.001	−0.044
Reoperation	19	0.00	29	0.01	26	0.01	0.002	−0.007
**Expenditures** (USD)								
Outpatient	14,580	34,986	61,139	58,262	91,170	78,268	<0.001	−1.263
Skilled nursing care	77,387	77,310	85,112	82,754	127,856	136,068	<0.001	−0.456
Inpatient (Surgery)	58,745	36,910	61,235	35,782	69,796	45,400	<0.001	−0.267
Readmission (30-day)^c^	29,621	51,007	23,297	39,661	30,139	45,078	<0.001	−0.011
Inpatient (31–365 days)^d^	82,609	108,626	85,896	113,917	110,094	148,527	<0.001	−0.211
Total	169,065	161,483	228,857	193,974	337,800	312,989	<0.001	−0.678

a Based on claims-based frailty index and categorized into robust (0–0.12), prefrail (0.12–0.20) and frail (>0.20).

b SMD for robust – frail.

c Readmission expenditures for patients with readmissions within 30 days.

d Inpatient expenditures for those with inpatient stays in this period Abbreviations: SD= Standard deviation, SMD= Standardized mean difference, SNF= Skilled nursing facility, DVT= Deep vein thrombosis, MI= Myocardial infarction, SSI= Surgical site infection, UTI= Urinary tract infection, USD= United States Dollars.

**Fig. 2:**
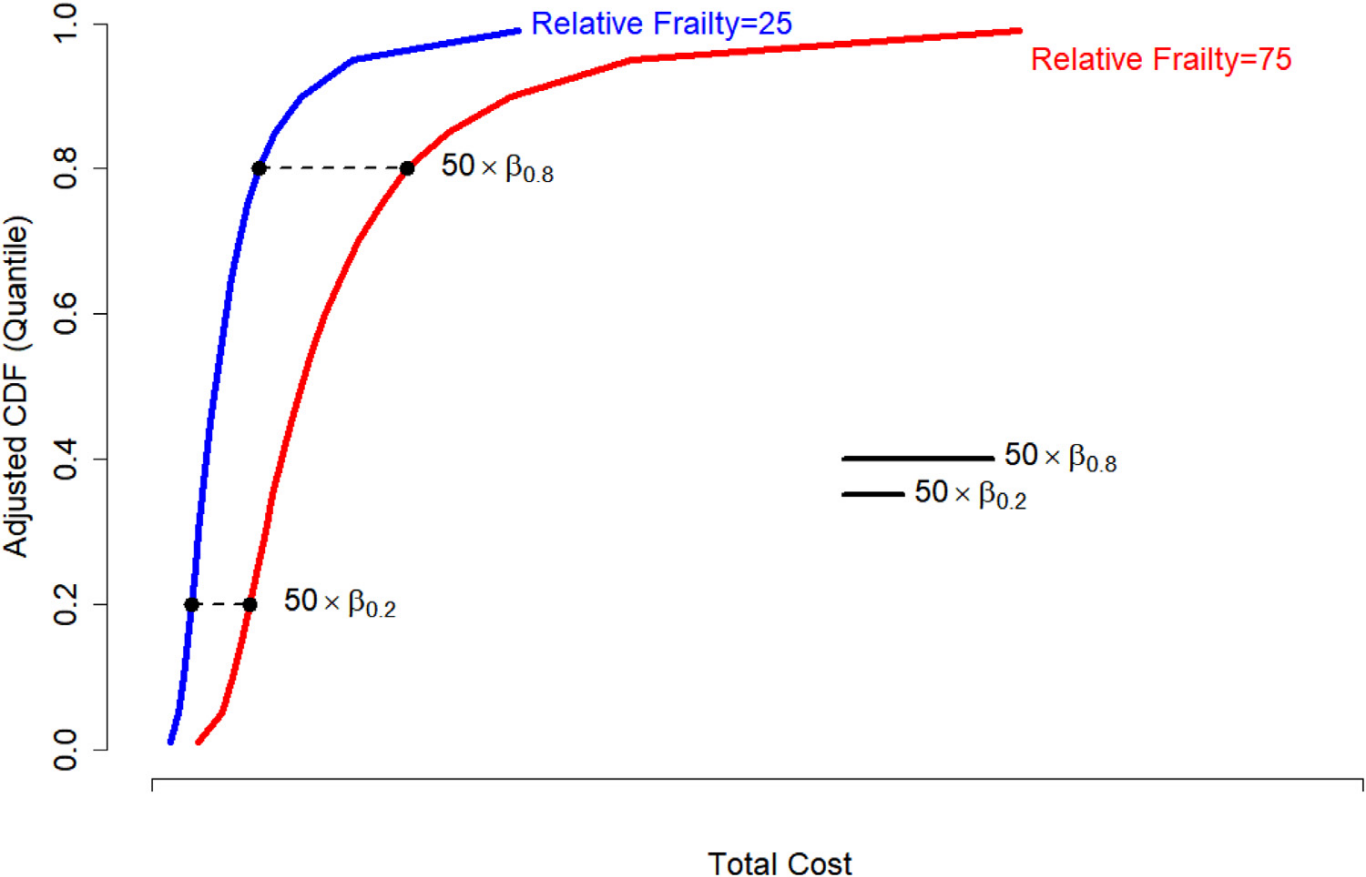
Difference in total expenditures at one year by relative frailty score Adjusted cumulative distribution function (CDF) for 2 different levels of relative frailty (solid blue and red lines), highlighting the differences at different quantile levels (dotted black lines). This figure illustrates that expenditures are higher among patients with greater relative degrees of frailty (red line) compared with those with lower relative frailty (blue line), and this difference becomes more pronounced as expenditures increase (black lines).

The unadjusted and adjusted beta estimates, which represent payment per unit of relative frailty, are presented in Table 3. We observed wide variation in the unadjusted beta estimates between the first, second and third quartiles for total payments at one year (beta [95 % CI]: Q1, 2,926 [2,910–2,942]; Q2, 4,004 [3,979–4,027]; Q3 6,246 [6,200–6,293]). These findings indicate that the differences in expenditures are not constant across quartiles and become larger as the expenditures increase. When we adjusted for patient and hospital characteristics, the wide variation in estimates persisted (beta [95 % CI]: Q1, 2,887 [2,871–2,903]; Q2, 3,970 [3,946–3,994]; Q3 6,143 [6,097–6,189]). Fig. 2 demonstrates the difference in expenditures depending on relative frailty score across different quantiles of cost.

**Table 3:** Estimated expenditures (in US dollars) per unit of relative frailty^a^.

	Unadjusted^b^		Adjusted^c^		HCC Adjusted^d^		
	Beta	95 % CI	Beta	95 % CI	Beta	95 % CI	p-value
Total expenditures							
Quartile 1	2,926	(2,910, 2,942)	1,767	(1,761, 1,773)	2,180	(2,163, 2,197)	<0.001
Quartile 2	4,004	(3,979, 4,028)	2,801	(2,795, 2,807)	2,492	(2,467, 2,516)	<0.001
Quartile 3	6,246	(6,200, 6,293)	3,143	(3,130, 3,156)	3,084	(3,042, 3,127)	<0.001
Outpatient							
Quartile 1	1,765	(1,759, 1,771)	565	(559, 571)	1,767	(1,761, 1,773)	<0.001
Quartile 2	2,979	(2,971, 2,987)	947	(937, 956)	2,710	(2,705, 2,715)	<0.001
Quartile 3	2,911	(2,898, 2,924)	1,770	(1,751, 1,790)	2,404	(2,391, 2,416)	<0.001
Skilled nursing care							
Quartile 1	597	(590, 603)	197	(193, 200)	279	(272, 285)	<0.001
Quartile 2	1,016	(1,006, 1,026)	274	(268, 279)	368	(357, 378)	<0.001
Quartile 3	1,895	(1,875, 1,915)	391	(383, 400)	502	(484, 521)	<0.001
Surgery							
Quartile 1	195	(191, 198)	88	(86, 91)	135	(131, 139)	<0.001
Quartile 2	282	(276, 288)	143	(138, 148)	155	(149, 162)	<0.001
Quartile 3	380	(371, 389)	219	(207, 231)	139	(129, 149)	<0.001
Readmission (1–30 days)^e^							
Quartile 1	96	(94, 99)	96	(83, 108)	27	(24, 30)	<0.001
Quartile 2	164	(160, 169)	456	(437, 476)	2	(−2, 7)	0.306
Quartile 3	265	(253, 277)	1,202	(1,160, 1,244)	−99	(−108, −90)	<0.001
Inpatient (31–365 days)^f^							
Quartile 1	66	(54, 78)	2,887	(2,871, 2,903)	−24	(−38, −9)	0.001
Quartile 2	457	(437, 477)	3,970	(3,946, 3,994)	43	(21, 65)	<0.001
Quartile 3	1,230	(1,186, 1,275)	6,143	(6,097, 6,189)	132	(91, 173)	<0.001

a Relative frailty (RF) was calculated by transforming the frailty index (scale 0–1) as follows: RF= 100×(Frailty - m)/(M - m) where m and M represent the minimum and maximum values in the cohort.

b First two p-value columns omitted to streamline table – all p-values for the unadjusted beta estimates <0.001.

c Adjusted for age, sex, race, Social Deprivation Index, surgery type, days alive, region and rural hospital. All p-values <0.001.

d Adjusted for all of the above plus Hierarchical Condition Category (HCC).

e Readmission expenditures for patients with readmissions within 30 days.

f Inpatient expenditures for those with inpatient stays 31–365 days after surgery.

**Fig. 3:**
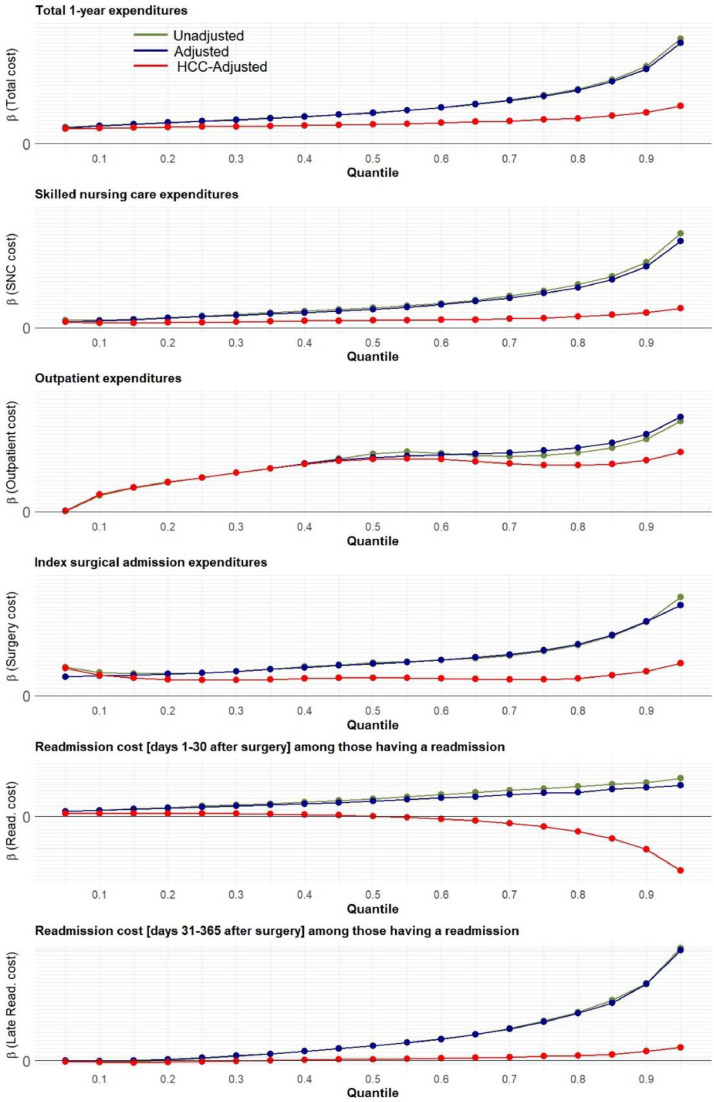
Unadjusted and adjusted quantile regression beta coefficients for the impact of relative frailty on expenditures at different quantiles of cost for total, skilled nursing, outpatient, surgery and readmission costs. This figure highlights the increasing beta coefficients across increasing cost quantiles, as well as the attenuation in the beta coefficients when adjusting for HCC score.

When adjusting for HCC, the variation in expenditures decreased, but remained significant. Fig. 3 shows the unadjusted, adjusted, and HCC adjusted quantile regression beta coefficients for the impact of relative frailty on expenditures at different levels of cost. The differences between the HCC adjusted and unadjusted models demonstrate that while payments are greater for patients with increasing degrees of frailty, adjusting for HCC attenuates these differences, suggesting that a large proportion of the difference in healthcare costs is anticipated based on the HCC.

### 3.3 Discharge destination, length of stay, readmissions, hospital complications and one-year mortality

Patients with frailty were less likely to be discharged to home compared with those who were prefrail or robust (n [%]: 126,515 [57.3]; 438,395 [80.2]; 314,221 [81.5]) (Table 2). For patients with frailty, alternative discharge locations included skilled nursing facilities (36,574 [16.6]), rehabilitation (14,962 [6.8]), and others (such as intermediate and long-term care facilities and acute care hospitals, 42,799 [19.4]). Length of stay was also longer in this group relative to the prefrail and robust groups (mean days [SD]: 2.8 [2.0], 2.2 [1.2], 2.1 [1.4], median [Q1-Q3]: 3.0 [2.0–3.0], 2.0 [1.0–3.0], 2.0 [1.0–3.0]). Readmission rates at 30 days were higher in those with frailty compared with those who were prefrail or robust (n [%]: 23,167 [10.5], 23,097 [4.2], 15,029 [3.9]). A similar pattern was seen for 90-day readmission rates (n [%]: 37,802 [17.1], 43,443 [8.0], 29,438 [7.6]). One-year mortality was also higher in those with frailty relative to the prefrail and robust groups (n [%]: 4,418 [1.2], 4,949 [0.9], 3,746 [1.0]).

Patients with frailty experienced higher rates of complications compared with those who were prefrail or robust. Notable complications included renal insufficiency (n [%]: 54,780 [24.8], 64,168 [11.8], 48,361 [12.5]), stroke (n [%]: 6,604 [3.0], 5,276 [1.0], 4,137 [1.1]), urinary tract infections (n [%]: 6,091 [2.8], 5,225 [1.0], 3,746 [1.0]), and delirium (n [%]: 3,126 [1.4], 615 [0.1], 1,142 [0.3]). A comprehensive list of hospital complications in addition to other secondary outcomes and unadjusted expenditures can be found in Table 2.

### 3.4 Evaluation of payment distribution (Gini index)

The Gini index (also known as Gini coefficient) is a measure of statistical dispersion traditionally used to quantify economic inequality on a scale of 0 to 1, with higher values indicating greater degrees of inequality. We used Gini coefficients to examine the degree of variation in expenditures among older adults undergoing hip and knee TJA. Among the cost subcategories, the category with the highest degree of inequality was 30-day readmission (Gini coefficient 0.903, 95 %CI 0.902, 0.903), although much of this inequality may be related to the fact that most patients were not readmitted. Outpatient expenditures also exhibited a high degree of inequality among beneficiaries (Gini coefficient 0.566, 95 %CI 0.565, 0.567), but variation in expenditures for the surgical admission (Gini coefficient 0.299, 95 %CI 0.298, 0.299) was lower than the overall variation in total 1-year expenditures (Gini coefficient 0.386, 95 %CI 0.385, 0.386). Gini coefficients by cost and frailty group are detailed in **Supplemental Table 2**.

### 3.5 Sensitivity analyses

When examining the association between frailty and expenditures separately among patients undergoing total hip and total knee arthroplasty, the resulting beta estimates exhibited similar trends of increasing costs with increasing degrees of frailty, as well as increasing variation in cost between more and less frail patients as costs increased. Hip arthroplasty presented slightly more pronounced cost variation compared with knee arthroplasty. See **Supplemental Tables 3–8** for the patient characteristics, expenditures, outcomes and beta coefficients separately for hip and knee surgeries.

## 4 Discussion

In this nationally representative sample of Medicare beneficiaries who underwent total hip or total knee arthroplasty, we found that increasing frailty was associated with greater expenditures in the year following the procedure. Frailty was also associated with longer lengths of stay, higher risks of complications, readmission, and mortality and lower likelihood of being discharged home after the procedure. Among older patients undergoing TJA procedures, the surgical procedure accounted for less than a third of the total 1-year healthcare expenditures, and was the cost category with the lowest degree of variation between patients with different degrees of frailty. These findings suggest that the increase in expenditures associated with frailty is predominantly related to longitudinal care over the course of the year following the procedure rather than to the primary surgical episode.

The differences in healthcare expenditures after TJA were greatly attenuated but remained significant when adjusting for HCC, indicating that arthroplasty is still associated with higher expenditures for frail patients even when adjusting for comorbidity burden. Although increasing frailty is associated with higher costs and increased risks of adverse outcomes after TJA, paradoxically, arthroplasty is often considered essential for frail patients to enable improved mobility and exercise capacity, which may in turn reduce frailty itself [11,15].

Our findings are in line with the results of other studies that have found that frailty is associated with numerous adverse patient-centered and systems-centered outcomes [9,[17], [18], [19],^24^,^25^]. Various strategies have been studied to prevent or reduce frailty and its impact on perioperative outcomes. Multidisciplinary geriatric surgery programs and orthogeriatric co-management appear to reduce postoperative length of stay, complications, and rehospitalization for older adults [[26], [27], [28], [29], [30], [31]]. Prehabilitation interventions for older adults may be able to reduce the burden of frailty preoperatively, and therefore decrease both adverse patient outcomes and economic burden postoperatively [32]. Interventions focused on improving transitional care, such as timely post-discharge primary care follow-up, may reduce postoperative readmission and mortality for older surgical patients [[33], [34], [35]]. Our results expose the characteristics and distribution of the added economic costs and healthcare utilization associated with frailty, suggesting that skilled nursing care, complications and readmission play a fundamental role. These findings indicate that implementation of programs that include multidisciplinary care, prehabilitation, and transitional care may prove cost-effective in addition to yielding more favorable functional and patient-centered outcomes for this patient population.

Medicare claims data are especially well suited for studying healthcare utilization among older adults since almost 99 % of the 65+ US population is covered by Medicare [36]. Using quantile regression to analyze payment data has several benefits. Importantly, it does not require the data to be normally distributed and does not assume equal spread of the data across quantiles. It also provides a more nuanced description of the added costs associated with frailty across the spectrum of cost levels. This more granular analysis provides additional insights, suggesting that the added cost of frailty is much more pronounced among patients with greater overall expenditures. In other words, as overall expenditures increase, the proportion of expenditures associated with frailty grows larger.

Subdividing the postoperative expenditures by category allowed us to examine the specific drivers of increased costs for patients with frailty, helping identify strategies to reduce costs. Our findings suggest that the most pronounced differences in cost between frailty groups are not related to the index hospitalization for surgery. In addition, as frailty increased, the surgical hospitalization accounted for a relatively smaller proportion of the total 1-year expenditures among Medicare beneficiaries who underwent arthroplasty procedures. Coupled with the fact that total joint arthroplasty has the potential to reduce frailty for older adults, these results imply that although healthcare expenditures are higher for more frail individuals, the overall cost effectiveness of TJA among frail patients is more nuanced and the potential functional outcomes need to be considered. Another important implication is that examining the financial consequences of frailty can help guide medical decisions and shape the development of value-based payment systems.

Pre-frail and frail older adults are increasingly undergoing elective TJA, and this procedure represents a major physiologic stressor for this patient population [37]. There is an urgent need to identify these patients in advance to prevent complications and manage costs associated with the failure to recognize their frailty prior to surgery [30,31]. It is no longer justifiable nor sustainable to treat older adults without preemptively assessing their robustness or frailty, as this oversight leads to missed opportunities for improving individual lives and healthcare systems by reducing adverse events [6,16,38].

### 4.1 Limitations

Since frailty index cut-off points for frailty categories continue to vary in the literature [[39], [40], [41]], we used data-driven cut points for frailty categories to describe our study cohort. The use of variable cut-off points across studies limits the ability to compare results between studies [42]. The reliance on the claims-based frailty index to define frailty also represents a limitation, as while it is a very useful tool for large scale databases, it does not include any physical performance measures. Another limitation is that our data included only Medicare expenditures and patients may have incurred other healthcare costs not paid by Medicare. In addition, owing to the limitations of Medicare claims data, we were unable to ascertain whether hospitals had implemented enhanced recovery protocols or orthogeriatric best practice. These practices could have differentially influenced both clinical and cost outcomes and would not have been entirely accounted for by adjusting for rurality or Social Deprivation Index.

## 5 Conclusions

Among older adults undergoing total hip and total knee arthroplasty, increasing degrees of frailty are associated with increasing healthcare expenditures, lengths of stay, complications, readmission, and mortality, and lower likelihood of discharge home after the procedure. Prehabilitation, transitional care, and multidisciplinary interventions that target individuals with frailty have the potential to reduce costs and improve outcomes for this patient population. More research is needed to confirm the cost-effectiveness and functional outcomes of such interventions for the benefit of older surgical patients, their caregivers, and healthcare systems.

## Conflicts of interest

On behalf of all authors, the corresponding author states that there is no conflict of interest.

## Appendix Supplementary materials

Image, application 1
