# Supplementary material for: Frailty and associated healthcare expenditures among patients undergoing total hip and knee arthroplasty
Source: J Frailty Aging. 2025 Mar 5;14(2):100030. doi: 10.1016/j.tjfa.2025.100030 (PMC12184003; doi:10.1016/j.tjfa.2025.100030)
Supplement: Supplementary file 1 [file mmc1.docx]

**Frailty and associated healthcare expenditures among patients undergoing total hip and knee arthroplasty**

Supplemental Materials

- Supplemental Table 1. Procedural codes for total hip and knee surgery
- Supplemental Figure 1. Inclusion flowchart
- Supplemental Table 2. Gini coefficients by cost and frailty group
- Supplemental Table 3. Baseline Patient Characteristics by Frailty – Total Hip Arthroplasty
- Supplemental Table 4. Baseline Patient Characteristics by Frailty – Total Knee Arthroplasty
- Supplemental Table 5. Frailty, Outcomes and Expenditures – total hip arthroplasty (unadjusted)
- Supplemental Table 6. Frailty, Outcomes and Expenditures – total knee arthroplasty (unadjusted)
- Supplemental Table 7. Estimated expenditures (in US dollars) per unit of relative frailty – total hip arthroplasty
- Supplemental Table 8. Estimated expenditures (in US dollars) per unit of relative frailty – total knee arthroplasty

**Supplemental Table 1. Procedural codes for total hip and knee surgery**

|  | **ICD-10^a^ Codes** |
| --- | --- |
| **Total Hip Arthroplasty** | 0SP90JZ, 0SPB0JZ, 0SR9019, 0SR901A, 0SR901Z, 0SR9029, 0SR902A, 0SR902Z, 0SR9039, 0SR903A, 0SR903Z, 0SR9049, 0SR904A, 0SR904Z, 0SR9069, 0SR906A, 0SR906Z, 0SR907Z, 0SR90EZ, 0SR90J9, 0SR90JA, 0SR90JZ, 0SR90KZ, 0SRB019, 0SRB01A, 0SRB01Z, 0SRB029, 0SRB02A, 0SRB02Z, 0SRB039, 0SRB03A, 0SRB03Z, 0SRB049, 0SRB04A, 0SRB04Z, 0SRB069, 0SRB06A, 0SRB06Z, 0SRB07Z, 0SRB0EZ, 0SRB0J9, 0SRB0JA, 0SRB0JZ, 0SRB0KZ, 0SW90JZ, 0SWB0JZ |
| **Total Knee Arthroplasty** | 0SPC0JZ, 0SPD0JZ, 0SRC069, 0SRC06A, 0SRC06Z, 0SRC07Z, 0SRC0EZ, 0SRC0J9, 0SRC0JA, 0SRC0JZ, 0SRC0M9, 0SRC0MA, 0SRC0MZ, 0SRC0N9, 0SRC0NA, 0SRC0NZ, 0SRD0EZ, 0SRD0M9, 0SRD0MA, 0SRD0MZ, 0SRD0N9, 0SRD0NA, 0SRD0NZ, 0SRC0KZ, 0SRD069, 0SRD06A, 0SRD06Z, 0SRD07Z, 0SRD0J9, 0SRD0JA, 0SRD0JZ, 0SRD0KZ, 0SRT07Z, 0SRT0J9, 0SRT0JA, 0SRT0JZ, 0SRT0KZ, 0SRU07Z, 0SRU0J9, 0SRU0JA, 0SRU0JZ, 0SRU0KZ, 0SRV07Z, 0SRV0J9, 0SRV0JA, 0SRV0JZ, 0SRV0KZ, 0SRW07Z, 0SRW0J9, 0SRW0JA, 0SRW0JZ, 0SRW0KZ, 0SWC0JZ, 0SWD0JZ |

1. International Statistical Classification of Diseases, 10th Revision, Clinical Modification (ICD-10-CM)

**Supplemental Figure 1. Inclusion flowchart**


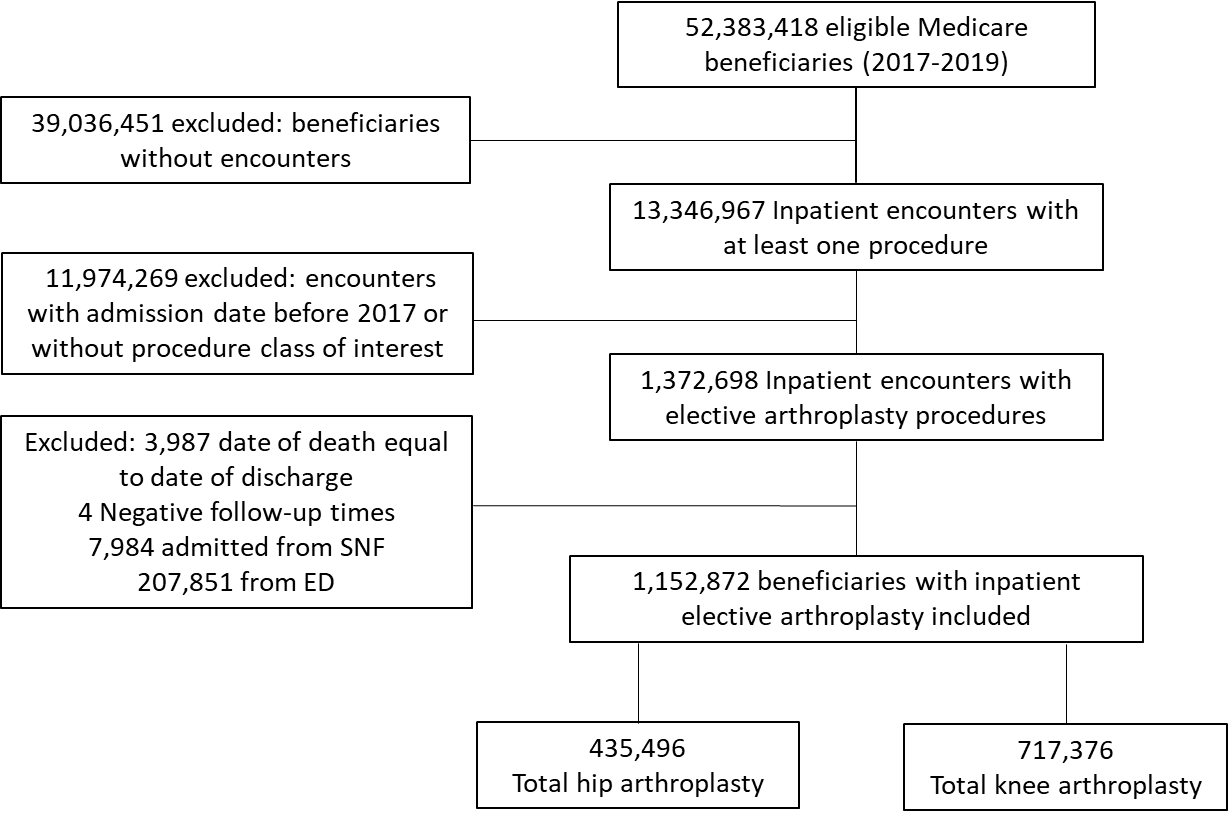


**Supplemental Table 2. Gini coefficients by cost and frailty group**

|  | **Total** | | | **Robust** | | | **Prefrail** | | | **Frail** | | |
| --- | --- | --- | --- | --- | --- | --- | --- | --- | --- | --- | --- | --- |
|  | **Gini** | **95% CI** | | **Gini** | **95% CI** | | **Gini** | **95% CI** | | **Gini** | **95% CI** | |
| **Outpatient** | 0.566 | 0.565 | 0.567 | 0.839 | 0.838 | 0.840 | 0.433 | 0.431 | 0.433 | 0.384 | 0.382 | 0.385 |
| **Skilled nursing care** | 0.406 | 0.406 | 0.407 | 0.392 | 0.391 | 0.394 | 0.378 | 0.377 | 0.379 | 0.431 | 0.429 | 0.432 |
| **Surgery** | 0.299 | 0.298 | 0.299 | 0.310 | 0.309 | 0.311 | 0.285 | 0.285 | 0.286 | 0.303 | 0.302 | 0.304 |
| **30-day Readmission** | 0.903 | 0.902 | 0.903 | 0.958 | 0.958 | 0.959 | 0.907 | 0.907 | 0.908 | 0.790 | 0.788 | 0.792 |
| **Inpatient cost (31-365 days)** | 0.892 | 0.891 | 0.893 | 0.904 | 0.904 | 0.905 | 0.899 | 0.898 | 0.899 | 0.847 | 0.846 | 0.849 |
| **Total expenditures** | 0.386 | 0.385 | 0.386 | 0.385 | 0.384 | 0.386 | 0.342 | 0.340 | 0.342 | 0.382 | 0.380 | 0.384 |

**Supplemental Table 3. Baseline Patient Characteristics by Frailty^a^ – Total Hip Arthroplasty**

|  | **Robust** (n=138,441) | | **Prefrail** (n=199,937) | | **Frail** (n=97,118) | |  |  |
| --- | --- | --- | --- | --- | --- | --- | --- | --- |
|  | N/mean | %/SD | N/mean | %/SD | N/mean | %/SD | p-value | SMD^b^ |
| **Frailty score** | 0.10 | 0.01 | 0.16 | 0.02 | 0.25 | 0.0 | <0.001 | -4.59 |
| **Age** in years | 73.8 | 6.2 | 73.4 | 6.3 | 76.8 | 7.3 | <0.001 | -0.44 |
| **Female** **sex** | 79,623 | 57.5 | 118,772 | 59.4 | 67,077 | 69.1 | <0.001 | -0.24 |
| **Race** |  |  |  |  |  |  |  |  |
| Asian | 661 | 0.5 | 831 | 0.4 | 364 | 0.4 | 0.001 | 0.02 |
| Black | 8,394 | 6.1 | 10,401 | 5.2 | 4,727 | 4.9 | <0.001 | 0.05 |
| Hispanic | 933 | 0.7 | 806 | 0.4 | 547 | 0.6 | <0.001 | 0.01 |
| North American Native | 177 | 0.1 | 550 | 0.3 | 387 | 0.4 | <0.001 | -0.05 |
| Other | 1,362 | 1.0 | 1,773 | 0.9 | 612 | 0.6 | <0.001 | 0.04 |
| Unknown | 3,327 | 2.4 | 4,959 | 2.5 | 981 | 1.0 | <0.001 | 0.11 |
| White | 123,587 | 89.3 | 180,617 | 90.3 | 89,500 | 92.2 | <0.001 | -0.10 |
| **Region** |  |  |  |  |  |  |  |  |
| Midwest | 41,632 | 30.1 | 64,142 | 32.1 | 31,861 | 32.8 | <0.001 | -0.06 |
| Northeast | 26,378 | 19.1 | 37,155 | 18.6 | 16,472 | 17.0 | <0.001 | 0.05 |
| South | 34,632 | 25.0 | 56,111 | 28.1 | 30,105 | 31.0 | <0.001 | -0.13 |
| West | 33,508 | 24.2 | 39,358 | 19.7 | 17,392 | 17.9 | <0.001 | 0.15 |
| Outside Continental US | 2,291 | 1.7 | 3,171 | 1.6 | 1,288 | 1.3 | <0.001 | 0.03 |
| **Rural hospital** | 8,669 | 6.3 | 18,726 | 9.4 | 9,548 | 9.8 | <0.001 | -0.13 |
| **Social Deprivation Index** | 37.9 | 25.9 | 36.1 | 25.3 | 39.4 | 25.7 | <0.001 | -0.06 |
| **HCC Score** | 0.42 | 0.73 | 0.51 | 0.76 | 1.35 | 1.3 | <0.001 | -0.87 |

1. Based on claims-based frailty index and categorized into robust (0-0.12), prefrail (0.12-0.20) and frail (>0.20)
2. SMD for robust – frail

Abbreviations: SD= Standard deviation, SMD= Standardized mean difference, HCC= Hierarchical Condition Category

**Supplemental Table 4. Baseline Patient Characteristics by Frailty^a^ – Total Knee Arthroplasty**

|  | **Robust**  (n= 247,259) | | **Prefrail**  (n= 346,385) | | **Frail** (n= 123,732) | |  |  |
| --- | --- | --- | --- | --- | --- | --- | --- | --- |
|  | N/mean | %/SD | N/mean | %/SD | N/mean | %/SD | p-value | SMD^b^ |
| **Frailty score** | 0.10 | 0.01 | 0.16 | 0.02 | 0.24 | 0.04 | <0.001 | -5.01 |
| **Age** in years | 73.2 | 5.6 | 73.0 | 5.8 | 75.1 | 6.3 | <0.001 | -0.33 |
| **Female** **sex** | 145,484 | 58.8 | 216,967 | 62.6 | 89,688 | 72.5 | <0.001 | -0.29 |
| **Race** |  |  |  |  |  |  |  |  |
| Asian | 2,739 | 1.1 | 3,856 | 1.1 | 1,110 | 0.9 | <0.001 | 0.02 |
| Black | 17,024 | 6.9 | 21,088 | 6.1 | 7,311 | 5.9 | <0.001 | 0.04 |
| Hispanic | 4,518 | 1.8 | 4,189 | 1.2 | 1,588 | 1.3 | <0.001 | 0.04 |
| North American Native | 494 | 0.2 | 1,580 | 0.5 | 680 | 0.5 | <0.001 | -0.06 |
| Other | 3,872 | 1.6 | 4,662 | 1.3 | 1,188 | 1.0 | <0.001 | 0.05 |
| Unknown | 5,016 | 2.0 | 7,091 | 2.0 | 1,346 | 1.1 | <0.001 | 0.08 |
| White | 213,596 | 86.4 | 303,919 | 87.7 | 110,509 | 89.3 | <0.001 | -0.09 |
| **Region** |  |  |  |  |  |  |  |  |
| Midwest | 75,779 | 30.6 | 116,856 | 33.7 | 40,646 | 32.9 | <0.001 | -0.05 |
| Northeast | 45,597 | 18.4 | 59,951 | 17.3 | 19,981 | 16.1 | <0.001 | 0.06 |
| South | 65,251 | 26.4 | 103,403 | 29.9 | 40,951 | 33.1 | <0.001 | -0.15 |
| West | 54,201 | 21.9 | 60,796 | 17.6 | 20,612 | 16.7 | <0.001 | 0.13 |
| Outside Continental US | 6,431 | 2.6 | 5,379 | 1.6 | 1,542 | 1.2 | <0.001 | 0.10 |
| **Rural hospital** | 18,778 | 7.6 | 38,628 | 11.2 | 13,992 | 11.3 | <0.001 | -0.13 |
| **Social Deprivation Index** | 39.8 | 26.7 | 38.2 | 25.9 | 41.1 | 26.1 | <0.001 | -0.05 |
| **HCC Score** | 0.32 | 0.57 | 0.45 | 0.66 | 1.07 | 1.10 | <0.001 | -0.86 |

1. Based on claims-based frailty index and categorized into robust (0-0.12), prefrail (0.12-0.20) and frail (>0.20)
2. SMD for robust – frail

Abbreviations: SD= Standard deviation, SMD= Standardized mean difference, HCC= Hierarchical Condition Category

**Supplemental Table 5. Frailty^a^, Outcomes and Expenditures – total hip arthroplasty (unadjusted)**

|  | **Robust** (n=138,441) | | **Prefrail** (n=199,937) | | **Frail** (n=97,118) | | | |
| --- | --- | --- | --- | --- | --- | --- | --- | --- |
|  | **N/mean** | **%/SD** | **N/mean** | **%/SD** | **N/mean** | **%/SD** | **p-value** | **SMD^b^** |
| **Outcomes** |  |  |  |  |  |  |  |  |
| Length of Stay (days) | 2.03 | 1.62 | 2.04 | 1.34 | 2.95 | 2.25 | <0.001 | -0.47 |
| 30-day Readmission | 6,085 | 4.4 | 8,712 | 4.4 | 11,520 | 11.9 | <0.001 | -0.28 |
| 90-day Readmission | 11,586 | 8.4 | 16,453 | 8.2 | 18,550 | 19.1 | <0.001 | -0.32 |
| 30-day Mortality | 320 | 0.2 | 284 | 0.1 | 800 | 0.8 | <0.001 | -0.08 |
| Mortality (1 year) | 2,332 | 1.7 | 2,405 | 1.2 | 5,418 | 5.6 | <0.001 | -0.21 |
| **ICU stay** | 4,130 | 3.0 | 5,071 | 2.5 | 6,361 | 6.5 | <0.001 | -0.17 |
| **Discharge destination** | | | | | | | |  |
| Home | 112,425 | 81.2 | 163,660 | 81.9 | 53,207 | 54.8 | <0.001 | 0.59 |
| SNF | 11,664 | 8.4 | 14,490 | 7.2 | 16,909 | 17.4 | <0.001 | -0.27 |
| Rehabilitation | 1,896 | 1.4 | 5,214 | 2.6 | 7,229 | 7.4 | <0.001 | -0.30 |
| Other | 12,456 | 9.0 | 16,573 | 8.3 | 19,773 | 20.4 | <0.001 | -0.33 |
| **Hospital Complications** | | | | | | | |  |
| Delirium | 538 | 0.4 | 232 | 0.1 | 1,591 | 1.6 | <0.001 | -0.13 |
| Pneumonia | 293 | 0.2 | 284 | 0.1 | 773 | 0.8 | <0.001 | -0.08 |
| Pulmonary Embolism | 143 | 0.1 | 164 | 0.1 | 190 | 0.2 | <0.001 | -0.02 |
| DVT | 292 | 0.2 | 331 | 0.2 | 422 | 0.4 | <0.001 | -0.04 |
| Stroke | 1,703 | 1.2 | 1,953 | 1.0 | 3,149 | 3.2 | <0.001 | -0.14 |
| Cardiac Arrest | 25 | 0.0 | 27 | 0.0 | 44 | 0.0 | <0.001 | -0.02 |
| Acute MI | 214 | 0.2 | 177 | 0.1 | 451 | 0.5 | <0.001 | -0.06 |
| Renal Insufficiency | 18,306 | 13.2 | 22,131 | 11.1 | 25,142 | 25.9 | <0.001 | -0.32 |
| SSI | 34 | 0.0 | 27 | 0.0 | 56 | 0.1 | <0.001 | -0.02 |
| UTI | 1,664 | 1.2 | 2,044 | 1.0 | 3,568 | 3.7 | <0.001 | -0.16 |
| Sepsis | 120 | 0.1 | 85 | 0.0 | 311 | 0.3 | <0.001 | -0.05 |
| **Expenditures** (USD) |  |  |  |  |  |  |  |  |
| Outpatient | 12,047 | 32,878 | 60,788 | 61,677 | 93,629 | 80,140 | <0.001 | -1.33 |
| Skilled nursing care | 79,716 | 83,037 | 86,566 | 85,227 | 136,188 | 141,358 | <0.001 | -0.49 |
| Inpatient (Surgery) | 59,680 | 37,454 | 62,179 | 36,395 | 71,948 | 47,600 | <0.001 | -0.29 |
| Readmission (30-day)^c^ | 35,264 | 56,086 | 26,842 | 42,832 | 34,165 | 48,705 | <0.001 | 0.02 |
| Inpatient (31-365 days)^d^ | 87,725 | 118,700 | 89,364 | 117,318 | 113,084 | 146,080 | <0.001 | -0.19 |
| Total | 171,259 | 171,775 | 231,440 | 200,618 | 355,452 | 324,360 | <0.001 | -0.71 |

1. Based on claims-based frailty index and categorized into robust (0-0.12), prefrail (0.12-0.20) and frail (>0.20)
2. SMD for robust –frail
3. Readmission expenditures for patients with readmissions within 30 days
4. Inpatient expenditures for those with inpatient stays in this period

Abbreviations: SD= Standard deviation, SMD= Standardized mean difference, SNF= Skilled nursing facility, DVT= Deep vein thrombosis, MI= Myocardial infarction, SSI= Surgical site infection, UTI= Urinary tract infection, USD= United States Dollars

**Supplemental Table 6. Frailty^a^, Outcomes and Expenditures – total knee arthroplasty (unadjusted)**

|  | **Robust** (n= 247,259) | | **Prefrail** (n= 346,385) | | **Frail** (n= 123,732) | | | |
| --- | --- | --- | --- | --- | --- | --- | --- | --- |
|  | **N/mean** | **%/SD** | **N/mean** | **%/SD** | **N/mean** | **%/SD** | **p-value** | **SMD^b^** |
| **Outcomes** |  |  |  |  |  |  |  |  |
| Length of Stay (days) | 2.1 | 1.3 | 2.2 | 1.2 | 2.7 | 1.7 | <0.001 | -0.40 |
| 30-day Readmission | 8,944 | 3.6 | 14,385 | 4.2 | 11,647 | 9.4 | <0.001 | -0.24 |
| 90-day Readmission | 17,852 | 7.2 | 26,990 | 7.8 | 19,252 | 15.6 | <0.001 | -0.26 |
| 30-day Mortality | 275 | 0.1 | 335 | 0.1 | 301 | 0.2 | <0.001 | -0.03 |
| Mortality (1 year) | 2,086 | 0.8 | 2,544 | 0.7 | 2,714 | 2.2 | <0.001 | -0.11 |
| **ICU stay** | 6,024 | 2.4 | 8,249 | 2.4 | 5,119 | 4.1 | <0.001 | -0.10 |
| **Discharge destination** | | | | | | | |  |
| Home | 201,796 | 81.6 | 274,735 | 79.3 | 73,308 | 59.2 | <0.001 | 0.51 |
| SNF | 19,617 | 7.9 | 27,854 | 8.0 | 19,665 | 15.9 | <0.001 | -0.25 |
| Rehabilitation | 4,164 | 1.7 | 10,797 | 3.1 | 7,733 | 6.2 | <0.001 | -0.24 |
| Other | 21,682 | 8.8 | 32,999 | 9.5 | 23,026 | 18.6 | <0.001 | -0.29 |
| **Hospital Complications** | | | | | | | |  |
| Delirium | 604 | 0.24 | 383 | 0.11 | 1,535 | 1.24 | <0.001 | -0.12 |
| Pneumonia | 375 | 0.15 | 546 | 0.16 | 688 | 0.56 | <0.001 | -0.07 |
| Pulmonary Embolism | 486 | 0.20 | 759 | 0.22 | 371 | 0.30 | <0.001 | -0.02 |
| DVT | 617 | 0.25 | 1,013 | 0.29 | 626 | 0.51 | <0.001 | -0.04 |
| Stroke | 2,434 | 0.98 | 3,323 | 0.96 | 3,455 | 2.79 | <0.001 | -0.13 |
| Cardiac Arrest | 37 | 0.01 | 57 | 0.02 | 50 | 0.04 | <0.001 | -0.02 |
| Acute MI | 195 | 0.08 | 261 | 0.08 | 307 | 0.25 | <0.001 | -0.04 |
| Renal Insufficiency | 30,055 | 12.2 | 42,037 | 12.1 | 29,638 | 24.0 | <0.001 | -0.31 |
| SSI | 49 | 0.02 | 61 | 0.02 | 62 | 0.05 | <0.001 | -0.02 |
| UTI | 2,082 | 0.84 | 3,181 | 0.92 | 2,523 | 2.04 | <0.001 | -0.10 |
| Sepsis | 120 | 0.05 | 129 | 0.04 | 198 | 0.16 | <0.001 | -0.03 |
| **Expenditures** (USD) |  |  |  |  |  |  |  |  |
| Outpatient | 15,998 | 36,035 | 61,342 | 56,195 | 89,240 | 76,713 | <0.001 | -1.22 |
| Skilled nursing care | 76,083 | 73,878 | 84,274 | 81,281 | 121,316 | 131,398 | <0.001 | -0.42 |
| Inpatient (Surgery) | 58,221 | 36,592 | 60,690 | 35,412 | 68,106 | 43,521 | <0.001 | -0.25 |
| Readmission (30-days)^c^ | 26,525 | 47,711 | 21,498 | 37,825 | 26,685 | 41,405 | <0.001 | 0.00 |
| Inpatient (31-365 days)^d^ | 79,751 | 102,457 | 83,970 | 111,937 | 107,611 | 150,486 | <0.001 | -0.22 |
| Total | 167,837 | 155,410 | 227,366 | 190,017 | 323,945 | 303,047 | <0.001 | -0.65 |

1. Based on claims-based frailty index and categorized into robust (0-0.12), prefrail (0.12-0.20) and frail (>0.20)
2. SMD for robust –frail
3. Readmission expenditures for patients with readmissions within 30 days
4. Inpatient expenditures for those with inpatient stays in this period

Abbreviations: SD= Standard deviation, SMD= Standardized mean difference, SNF= Skilled nursing facility, DVT= Deep vein thrombosis, MI= Myocardial infarction, SSI= Surgical site infection, UTI= Urinary tract infection, USD= United States Dollars

**Supplemental Table 7.** **Estimated expenditures (in US dollars) per unit of relative frailty^a^ – total hip arthroplasty**

|  | Unadjusted | | | HCC Adjusted^b^ | | |
| --- | --- | --- | --- | --- | --- | --- |
|  | Beta | 95% CI | | Beta | 95% CI | |
| Total expenditures |  |  |  |  |  |  |
| Quartile 1 | 1,700 | 1,691 | 1,710 | 1,708 | 1,698 | 1,717 |
| Quartile 2 | 2,845 | 2,833 | 2,856 | 2,711 | 2,701 | 2,721 |
| Quartile 3 | 3,182 | 3,160 | 3,203 | 3,394 | 3,372 | 3,415 |
| Outpatient |  |  |  |  |  |  |
| Quartile 1 | 671 | 661 | 682 | 611 | 601 | 621 |
| Quartile 2 | 1,193 | 1,177 | 1,210 | 1,067 | 1,051 | 1,083 |
| Quartile 3 | 2,196 | 2,162 | 2,229 | 1,976 | 1,943 | 2,008 |
| Skilled nursing care |  |  |  |  |  |  |
| Quartile 1 | 214 | 208 | 220 | 212 | 206 | 218 |
| Quartile 2 | 318 | 309 | 327 | 298 | 290 | 306 |
| Quartile 3 | 433 | 418 | 448 | 425 | 411 | 439 |
| Surgery |  |  |  |  |  |  |
| Quartile 1 | 121 | 116 | 125 | 109 | 104 | 113 |
| Quartile 2 | 207 | 198 | 215 | 177 | 169 | 186 |
| Quartile 3 | 270 | 246 | 293 | 249 | 228 | 271 |
| Readmission (1-30 days)^c^ |  |  |  |  |  |  |
| Quartile 1 | 70 | 51 | 89 | 120 | 100 | 140 |
| Quartile 2 | 455 | 423 | 486 | 480 | 449 | 510 |
| Quartile 3 | 1,213 | 1,143 | 1,284 | 1,216 | 1,149 | 1,283 |
| Inpatient (31-365 days)^d^ |  |  |  |  |  |  |
| Quartile 1 | 3,051 | 3,025 | 3,077 | 2,974 | 2,949 | 3,000 |
| Quartile 2 | 4,334 | 4,295 | 4,373 | 4,207 | 4,168 | 4,246 |
| Quartile 3 | 6,848 | 6,771 | 6,925 | 6,616 | 6,544 | 6,688 |

1. Relative frailty (RF) was calculated by transforming the frailty index (scale 0-1) as follows: RF= 100×(Frailty - m)/(M - m) where m and M represent the minimum and maximum values in the cohort
2. Adjusted for age, sex, race, Social Deprivation Index, Hierarchical Condition Category (HCC), surgery type, days alive, region and rural hospital.
3. Readmission expenditures for patients with readmissions within 30 days.
4. Inpatient expenditures for those with inpatient stays 31-365 days after surgery.

**Supplemental Table 8. Estimated expenditures (in US dollars) per unit of relative frailty^a^ – total knee arthroplasty**

|  | **Unadjusted** | | | **HCC Adjusted^b^** | | |
| --- | --- | --- | --- | --- | --- | --- |
|  | Beta | 95% CI | | Beta | 95% CI | |
| **Total expenditures** |  |  |  |  |  |  |
| Quartile 1 | 2,835 | 2,814 | 2,856 | 2,821 | 2,800 | 2,842 |
| Quartile 2 | 3,771 | 3,740 | 3,802 | 3,796 | 3,766 | 3,826 |
| Quartile 3 | 5,775 | 5,715 | 5,835 | 5,798 | 5,741 | 5,855 |
| **Outpatient** |  |  |  |  |  |  |
| Quartile 1 | 1,820 | 1,811 | 1,828 | 1,820 | 1,811 | 1,828 |
| Quartile 2 | 3,073 | 3,061 | 3,084 | 2,882 | 2,874 | 2,890 |
| Quartile 3 | 2,764 | 2,747 | 2,780 | 2,965 | 2,949 | 2,981 |
| **Skilled nursing care** |  |  |  |  |  |  |
| Quartile 1 | 542 | 533 | 550 | 530 | 522 | 538 |
| Quartile 2 | 893 | 881 | 905 | 864 | 852 | 876 |
| Quartile 3 | 1,657 | 1,631 | 1,682 | 1,622 | 1,598 | 1,646 |
| **Surgery** |  |  |  |  |  |  |
| Quartile 1 | 174 | 170 | 179 | 182 | 177 | 187 |
| Quartile 2 | 250 | 242 | 258 | 254 | 248 | 261 |
| Quartile 3 | 330 | 318 | 343 | 364 | 353 | 376 |
| **Readmission (1-30 days)^c^** | | |  |  |  |  |
| Quartile 1 | 74 | 71 | 77 | 75 | 71 | 78 |
| Quartile 2 | 120 | 114 | 125 | 121 | 116 | 127 |
| Quartile 3 | 187 | 173 | 202 | 201 | 187 | 215 |
| **Inpatient (31-365 days)^d^** | |  |  |  |  |  |
| Quartile 1 | 58 | 43 | 74 | 82 | 66 | 98 |
| Quartile 2 | 438 | 413 | 464 | 447 | 423 | 471 |
| Quartile 3 | 1,187 | 1,129 | 1,245 | 1,191 | 1,137 | 1,245 |

1. Relative frailty (RF) was calculated by transforming the frailty index (scale 0-1) as follows: RF= 100×(Frailty - m)/(M - m) where m and M represent the minimum and maximum values in the cohort
2. Adjusted for age, sex, race, Social Deprivation Index, Hierarchical Condition Category (HCC), surgery type, days alive, region and rural hospital.
3. Readmission expenditures for patients with readmissions within 30 days.
4. Inpatient expenditures for those with inpatient stays 31-365 days after surgery.
